# Rapidly developed squamous cell carcinoma after laser therapy used to treat chemical burn wound: a case report

**DOI:** 10.1186/s12957-015-0437-5

**Published:** 2015-02-07

**Authors:** Hyung-Rok Cho, Soon-Sung Kwon, Seum Chung, Jeong-Hae Kie

**Affiliations:** Department of Plastic Surgery, National Health Insurance Service Ilsan Hospital, Gyeonggi, South Korea; Department of Pathology, National Health Insurance Service Ilsan Hospital, Gyeonggi, Korea

**Keywords:** Chemical burn, HeNe laser, Sodium hypochlorite, Squamous cell carcinoma

## Abstract

**Background:**

In chronic wounds, especially burn scars, malignant tumors can arise. However, it is rare for a subacute burn injury to change to a malignant lesion within one month. Moreover, a case of squamous cell carcinoma arising from HeNe laser therapy after a chemical burn has never been reported.

**Case report:**

In this report, we examine a rare case of squamous cell carcinoma arising from HeNe laser therapy after a chemical burn. Because pathologic investigations were made from the first operation, both early detection of the squamous cell carcinoma and consideration of the HeNe laser therapy as a risk factor for the skin cancer were possible. The cancer was completely removed and reconstruction of the defect was successfully achieved in a timely manner.

**Conclusion:**

Although there has as yet been no reported case of squamous cell carcinoma induced by laser therapy, it is important for clinicians to recognize both the possibility of laser-induced cancer and the rapid change of cancer, so they can provide appropriate and timely treatment.

## Background

Malignant tumors can arise from chronic scars, called Marjolin’s ulcers and, of these malignant tumors, the squamous cell carcinoma is the most common [1]. Although Marjolin’s ulcers can occur within 1 year of an injury, the average latency period from an injury to a cancer is reported as 31 years [2]. In wound healing, low-energy lasers, mainly helium-neon (HeNe) lasers, are known to be helpful to stimulate tissue regeneration [3,4]. Plastic surgeons do not routinely use HeNe lasers to manage burn wounds but, in local clinics, general physicians or dermatologists sometimes use them as the treatment modality. We examine the first case of squamous cell carcinoma arising within 1 month of HeNe laser therapy for a chemical burn. Because we asked the pathologist to investigate the tissue specimen from a first operation, early detection of squamous cell carcinoma was possible. After wide excision in a second operation, the malignant tumor was totally removed and the defect was successfully reconstructed.

## Case presentation

**Figure 1:**
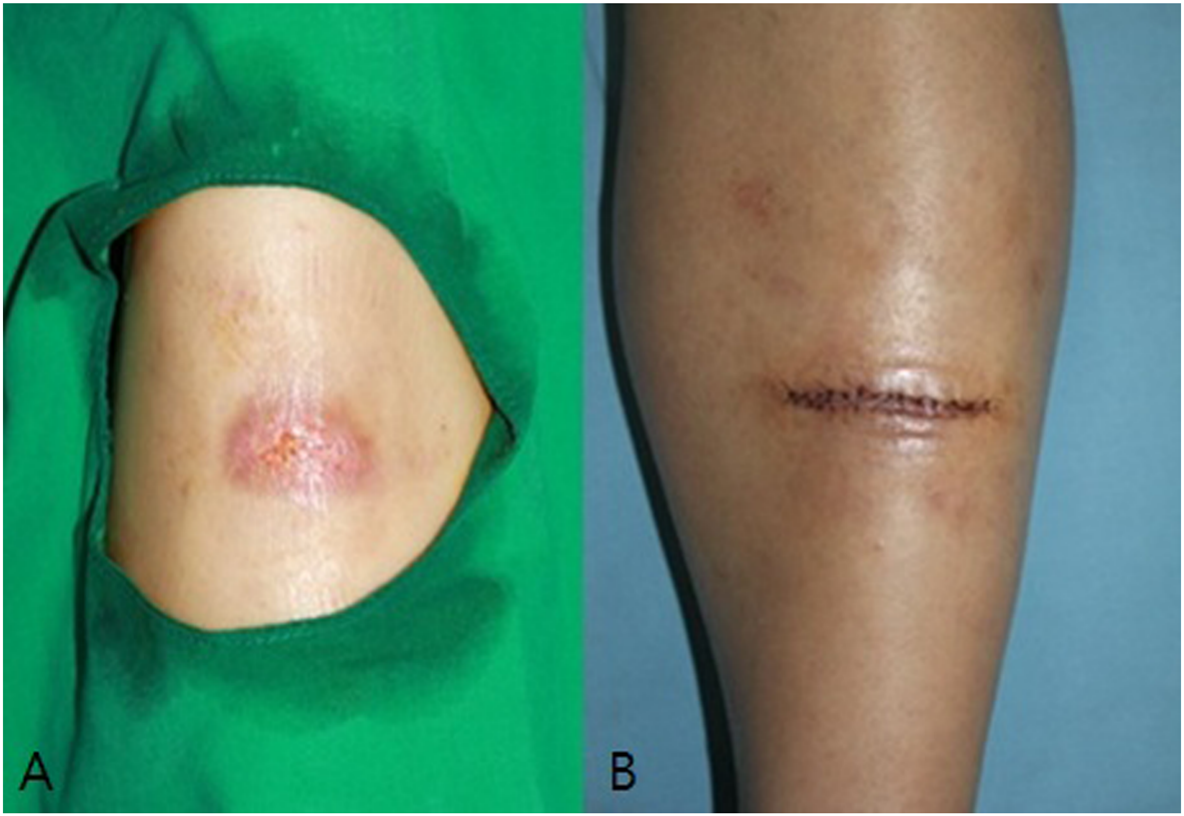
**Preoperative and postoperative images of the first operation. (A)** Open wound on left lower leg, about 3.5 × 1.5 cm^2^. There was no sign of infection or bleeding. **(B)** After excision, primary repair was performed.

A 53-year-old woman presented with an open wound on the left lower leg. It was a de-epithelized abrasion and its size was about 3.5 × 1.5 cm^2^ (Figure 1A). The patient had no notable medical history. She works as a janitor, and was cleaning the floor of the building, when she spilled undiluted cleansing solution that consisted of sodium hypochlorite (NaClO) on her left lower leg. After the accident, an abrasion was noted on that area and she visited her local dermatologic clinic for treatment on the very next day. At the clinic, she received HeNe laser therapy directly to the wound daily for 10 days. The treatment continued for 8 to 9 minutes on each occasion. However, we were unable to determine the details of laser therapy, such as power, wavelength, and so on. The treatment was not beneficial and the wound size was constant. The patient visited our clinic three weeks after the injury and we planned an excision of the unhealed area under the local anesthesia.

**Figure 2:**
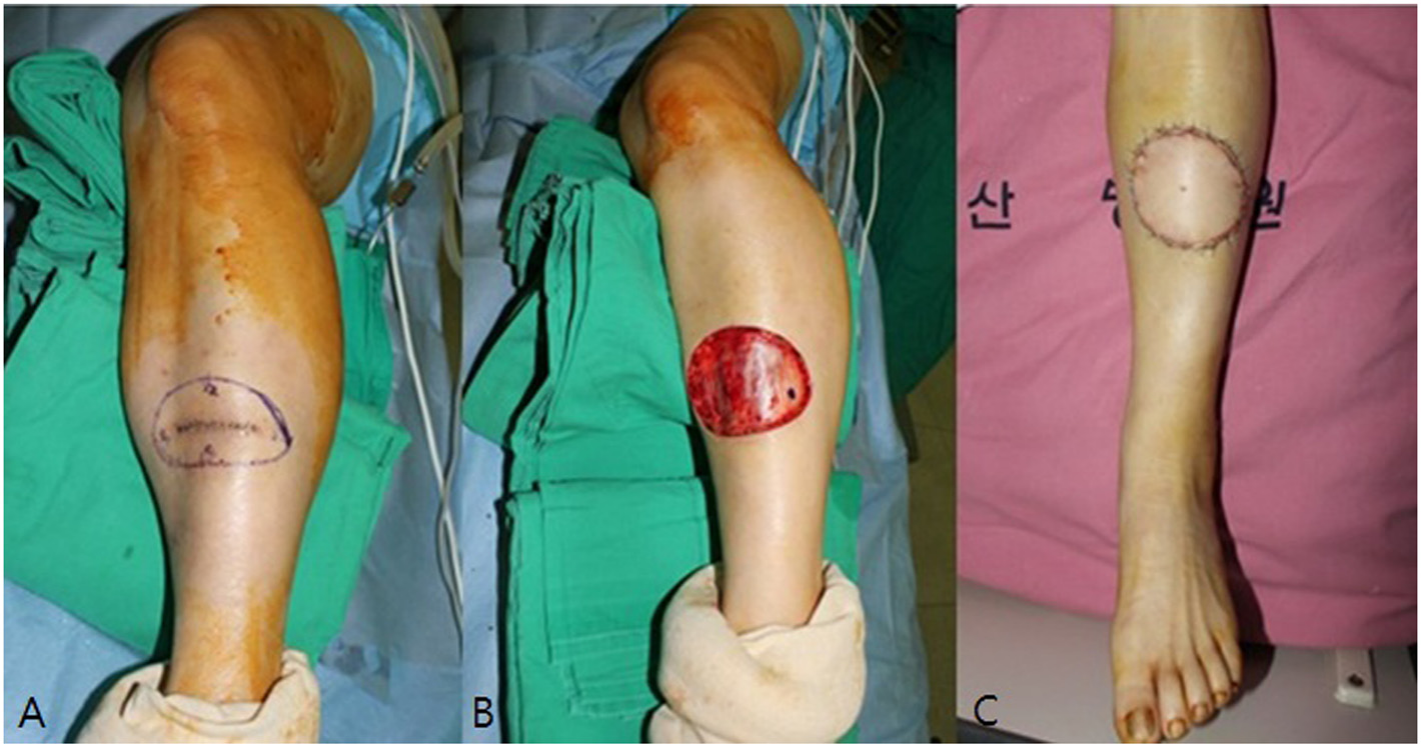
**Preoperative and postoperative images of the second operation. (A)** The incision line was drawn to allow a 2 to 3 cm safe margin from the tumor. **(B)** After wide excision, the size of the defect on the left lower leg was 9 × 7 cm^2^). The wound bed was deep fascia. **(C)** The defect was reconstructed with a left thoracodorsal artery perforator free flap. Microanastomosis was achieved, using the anterior tibial artery and its accompanying vein as recipient vessels.

**Figure 3:**
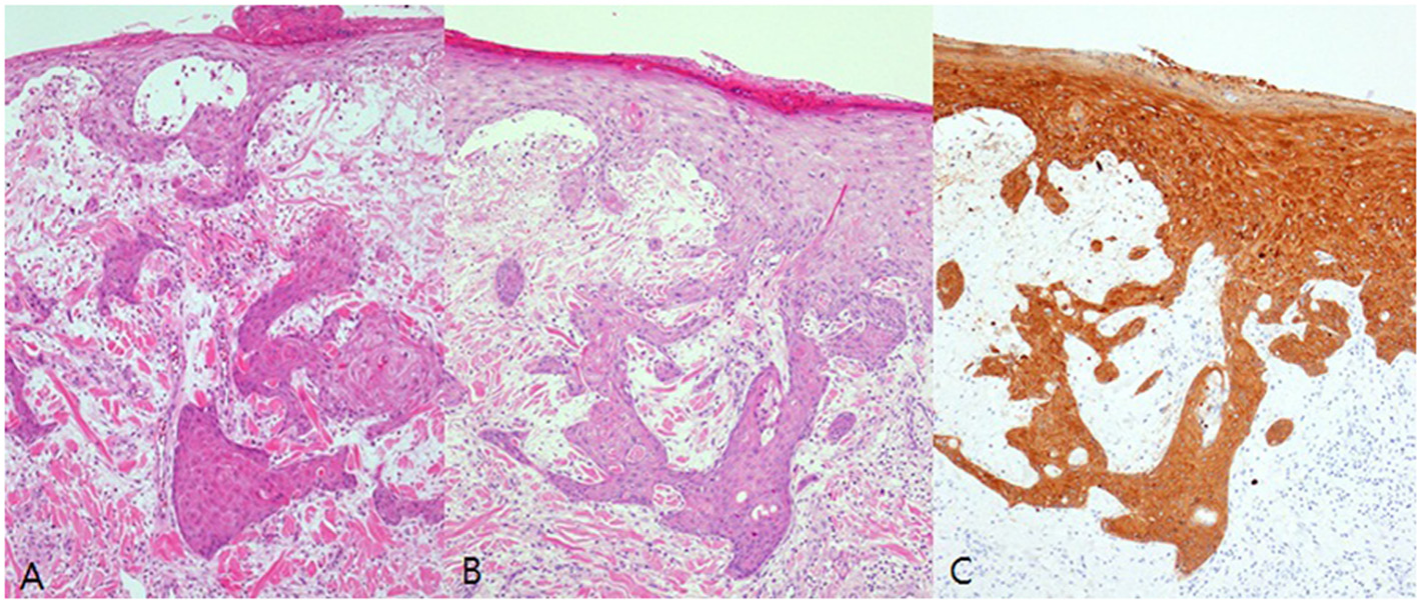
**H & E and CK5/6 staining of frozen section of tumor (×100). (A)** H & E stain. Well differentiated squamous cell carcinoma with marked foreign body reaction. A keratin pearl was noted. **(B)** H & E stain. **(C)** CK5/6 stain.

**Figure 4:**
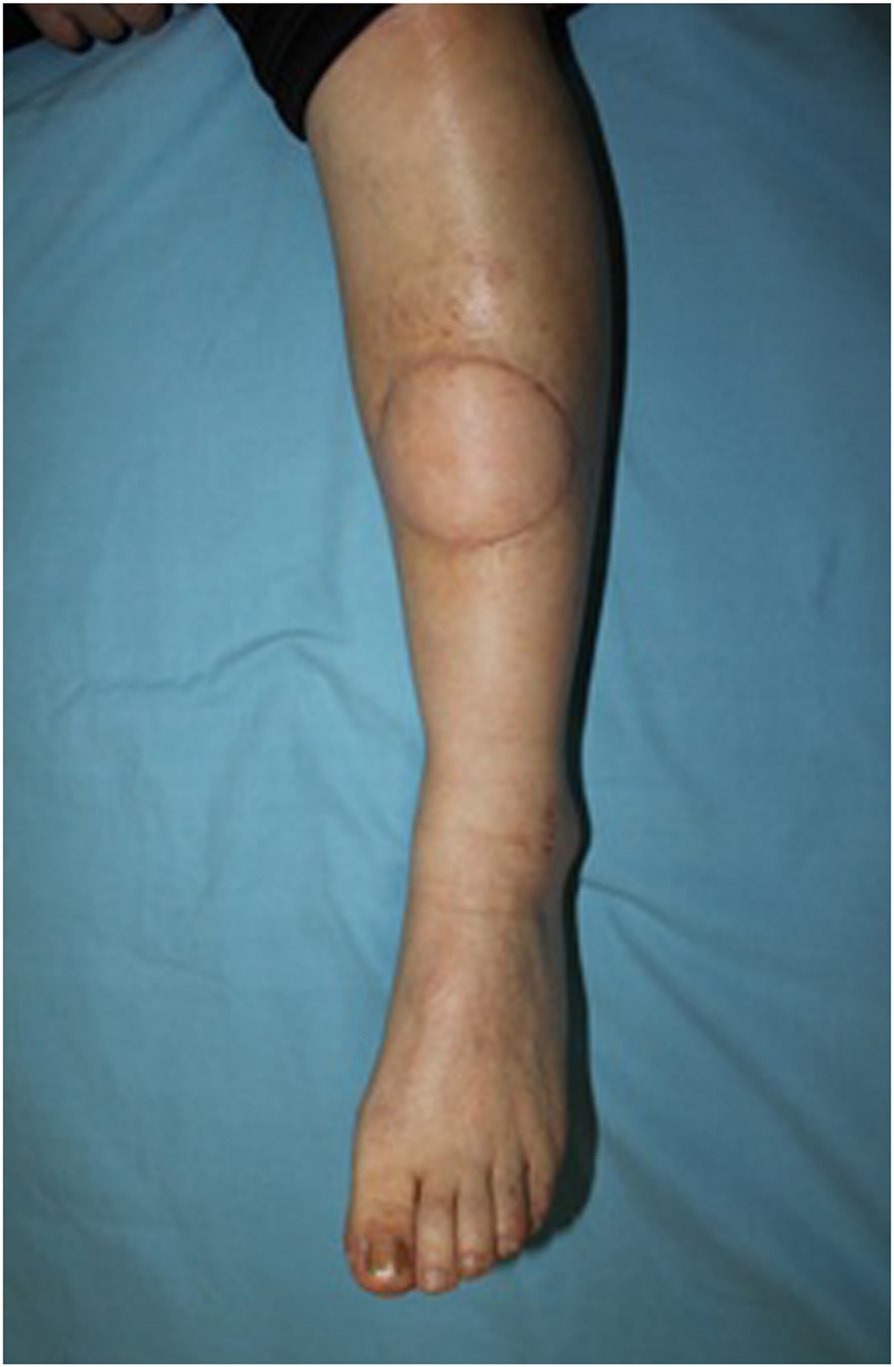
**3 months after surgery.** The thoracodorsal artery perforator flap was well taken with a good contour and there were no complications.

During the first operation, the open wound was excised and primary repair was done (Figure 1B). We sent a specimen to the pathology department and the pathologic analysis revealed invasive squamous cell carcinoma, 2 × 1.5 cm^2^ in size, involving the lateral resection margin. We planned a second operation and evaluated positron emission tomography–computed tomography for metastatic lesions before the second operation. After confirming that there was no metastasis, the patient received a second operation 3 weeks from the first. Wide excision was done with a 2 to 3 cm safe margin (Figure 2A). During the operation, the resection margin including the tumor base was free from tumors, as demonstrated by a frozen study. The total defect size of the left lower leg measured 9 × 7 cm^2^ and the defect was successfully reconstructed with a thoracodorsal artery perforator free flap transfer (Figure 2B,C). Microanastomosis was achieved, using the anterior tibial artery and its accompanying vein as recipient vessels. Permanent pathologic analysis revealed microscopic focal residual squamous cell carcinoma with marked foreign body reaction (Figure 3). The patient recovered without complication. After 3 months, the transferred flap was well taken with a good contour (Figure 4).

## Discussion

In chronic wounds, especially burn scars, malignant tumors can arise, called Marjolin’s ulcers, a long time after injury [1,2]. The most common type is squamous cell carcinoma. Although squamous cell carcinoma was diagnosed in this case, it is quite different from other regular cases. In this case, the malignant tumor arose from the wound within one month. It is true that Marjolin’s ulcers can occur within 1 year, but there was too rapid and early progression in this case. Moreover, it is usually thought that repeated trauma and prolonged healing are the risk factors for Marjolin’s ulcers [5,6]. After the accident, there was no other traumatic event and our patient had only HeNe laser therapy with a simple dressing.

In this case, the patient was injured by undiluted sodium hypochlorite, which is a widely known rapid and effective antimicrobial [7]. It is also usually used by cleaners as a cleansing solution. Although this solution is known to produce no significant damage to intact skin, concentrated sodium hypochlorite can have a cytotoxic effect on human cells [8,9]. Our patient spilled an undiluted solution, which is recommended to use after diluting 300 times with water. The high concentration exposure caused a chemical burn on the patient’s left lower leg. Abrasion has been observed frequently where patients have contact with this solution. However, the skin usually recovers within several days with a simple dressing. It is rare for a superficial chemical burn wound caused by sodium hypochlorite solution to change to a malignant lesion, although it was not as deep as a Marjolin’s ulcer.

The low-power HeNe laser used in this case has the effect of modulating tissue metabolism. It can stimulate wound healing and tissue regeneration by increasing collagen production, promoting new vessel formation and stimulating epithelization [10,11]. In our case, the patient had had HeNe laser therapy daily for 10 days. It has never been reported that low-energy HeNe laser treatment can cause skin cancer. Although it is not the only cause of the malignancy, it might be one of the factors that caused the burn wound to change to a malignant lesion.

It could also be that a previous skin cancer was accidentally injured by the chemical burn. However, the patient insisted that there was no previous skin lesion on her left lower leg. Furthermore, pathologic analysis of the specimen from the first operation revealed that the tumor size was not that small and that the margin was infiltrated by the squamous cell carcinoma. It is more likely that the burn wound changed to the malignancy rather than that a previous skin cancer existed.

Fortunately, by our pathologic investigation the unhealed wound, we could detect the skin cancer promptly. The patient had a proper radiologic evaluation and there was no metastatic lesion. Wide excision of the skin cancer was performed and the defect was successfully reconstructed without any morbidity. In this case, we used a thoracodorsal artery perforator free flap for reconstructing the defect. We mainly considered aesthetic and functional reconstruction. Most surgeons might choose a skin graft because of the simplicity of the technique and early detection of local recurrence. However, it is very unaesthetic to use a skin graft in reconstructing the female lower leg and a skin graft would also be unstable because of muscle movement. Therefore, we used a free flap and the patient was satisfied with the outcome.

## Conclusions

We have reported a rare case of squamous cell carcinoma from laser therapy after a chemical burn wound. We insist that it is important for clinicians to perform a skin biopsy on a suspicious wound and recognize both the possibility of laser-induced cancer and a rapid change of cancer, so they can provide appropriate and timely treatment. We also recommend further studies on the effects of the laser on the skin, regarding its possible causation of skin cancer.

## Consent

Written informed consent was obtained from the patient for publication of this manuscript and any accompanying images. A copy of the written consent is available for review by the Editor-in-Chief of this journal.

## Abbreviations

H & E: Hematoxylin and eosin
HeNe: Helium-neon

## References

[1] Treves N, Pack GT (1930). The development of cancer in burn scars: an analysis and report of thirty-four cases. Surg Gynecol Obstet.

[2] Kowal-Vern A, Criswell BK (2005). Burn scar neoplasms: a literature review and statistical analysis. Burns..

[3] Kana JS, Hutschenreiter G, Haina D, Waidelich W (1981). Effect of low-power density laser radiation on healing of open skin wounds in rats. Arch Surg.

[4] Lyons RF, Abergel RP, White RA, Dwyer RM, Castel JC, Uitto J (1987). Biostimulation of wound healing in vivo by a helium neon laser. Ann Plast Surg.

[5] Edwards MJ, Hirsch RM, Broadwater JR, Netscher DT, Ames FC (1989). Squamous cell carcinoma arising in previously burned or irradiated skin. Arch Surg..

[6] Ozek C, Cankayali R, Bilkay U, Guner U, Gundogan H, Sonqur E (2001). Marjolin’s ulcers arising in burn scars. J Burn Care Rehabil..

[7] Rutala WA, Weber DJ (1997). Uses of inorganic hypochlorite (bleach) in health-care facilities. Clin. Microbiol. Rev..

[8] McKenna SM, Davies KJA (1988). The inhibition of bacterial growth by hypochlorous acid. Possible role in the bactericidal activity of phagocytes. Biochem J.

[9] Hidalgo E, Bartolome R, Dominguez C (2002). Cytotoxicity mechanisms of sodium hypochlorite in cultured human dermal fibroblasts and its bactericidal effectiveness. Chem Biol Interact.

[10] Mester E, Toth N, Mester A (1982). The biostimulative effect of laserbeam. Laser Basic Biomed Res..

[11] Js S, Alago ML, Bellamy RF, Stuck BE, Belkin M (1983). Effects of low-level energy lasers on the healing of full-thickness skin defects. Lasers Surg Med.

